# RpoS Regulates Essential Virulence Factors Remaining to Be Identified in *Borrelia burgdorferi*

**DOI:** 10.1371/journal.pone.0053212

**Published:** 2012-12-27

**Authors:** Qilong Xu, Yanlin Shi, Poonam Dadhwal, Fang Ting Liang

**Affiliations:** Department of Pathobiological Sciences, Louisiana State University, Baton Rouge, Louisiana, United States of America; University of Kentucky College of Medicine, United States of America

## Abstract

**Background:**

Since the RpoN-RpoS regulatory network was revealed in the Lyme disease spirochete *Borrelia burgdorferi* a decade ago, both upstream and downstream of the pathway have been intensively investigated. While significant progress has been made into understanding of how the network is regulated, most notably, discovering a relationship of the network with Rrp2 and BosR, only three crucial virulence factors, including outer surface protein C (OspC) and decorin-binding proteins (Dbps) A and B, are associated with the pathway. Moreover, for more than 10 years no single RpoS-controlled gene has been found to be critical for infection, raising a question about whether additional RpoS-dependent virulence factors remain to be identified.

**Methodology/Principal Findings:**

The *rpoS* gene was deleted in *B. burgdorferi*; resulting mutants were modified to constitutively express all the known virulence factors, OspC, DbpA and DbpB. This genetic modification was unable to restore the *rpoS* mutant with infectivity.

**Conclusions/Significance:**

The inability to restore the *rpoS* mutant with infectivity by simultaneously over-expressing all the three virulence factors allows us to conclude RpoS also regulates essential genes that remain to be identified in *B. burgdorferi*.

## Introduction

The Lyme disease spirochete *Borrelia burgdorferi* has three *σ* factors, including the major factor, RpoD (*σ* ^70^), and two alternative factors, RpoN (*σ* ^54^) and RpoS (*σ* ^38^). A pioneering study by Norgard and colleagues published in 2001 successfully associated the two alternative factors to form a regulatory network, in which RpoS expression depends on RpoN and controls expression of at least three important surface lipoproteins including outer surface protein C (OspC), and decorin-binding proteins (Dbps) A and B [1], This started the era of intensively investigating both up- and down-stream of the RpoN-RpoS pathway [2]. Yang *et al.* promptly added the response regulator Rrp2 to the pathway, in which Rrp2 controls the activity of RpoN, which in turn controls RpoS expression [3]. Recently Yang and colleagues further showed Rrp2 can simply be activated by acetyl-phosphate [4]. Another line of study by at least two independent groups showed BosR is essential for expression of RpoS [5]–[8]. A third line of investigation led by Samuels showed small RNA is also involved in regulation of RpoS [9]. These studies have significantly advanced understanding of how the RpoN-RpoS network is regulated but also highlight the complicated nature of the network.

In contrast to significant progress made towards understanding of the upstream regulation of the RpoN-RpoS pathway, very little information has been gained regarding virulence genes regulated by the pathway since initial identification of the network. Fisher *et al.* by using microarray analysis showed RpoN and RpoS can either independently regulate many genes or form an RpoN-RpoS regulatory cascade to regulate even more genes [10]. By using the same technique Radolf and colleagues identified 137 genes, whose expression is differentially regulated by RpoS [11]. While this screening technique was unable to expand knowledge on the spectrum of virulence genes controlled by RpoS, when careful studies focusing on an individual gene was conducted, several genes, including *bba64*, *bb0844*, *bba07*and *bbk32* (fibronectin-binding protein gene have been confirmed to be RpoS-dependent [12]–[14] since the RpoN-RpoS pathway was discovered more than a decade ago [1]. Among these newly identified RpoS-regulated factors, only BBA07 and BBK32 were shown some roles in mammalian infection [13], [15]–[17].

No new crucial virulence factor controlled by RpoS has been identified after 10 years of intensive investigation, raising a question about whether the RpoN-RpoS pathway also regulates additional important genes. As deletion of the *ospC* gene alone abrogates infectivity, the essential role of RpoS in mammalian infection can be simply explained as its role in controlling OspC expression [18]. Moreover, lack of either DbpA or DbpB dramatically reduces infectivity, and deletion of both causes an accumulative effect [19]–[22]. To examine whether RpoS regulates essential genes that remain to be identified, the *rpoS* gene was first deleted. A resulting mutant was then modified to constitutively express *ospC*, *dbpA* and *dbpB*, and inoculated into mice. The inability to restore infectivity by simultaneously over-expressing the three crucial virulence factors indicated that other essential gene(s) controlled by the RpoN-RpoS pathway remains to be identified.

## Materials and Methods

### Construction of disruption plasmid pSKO

**Figure 1:**
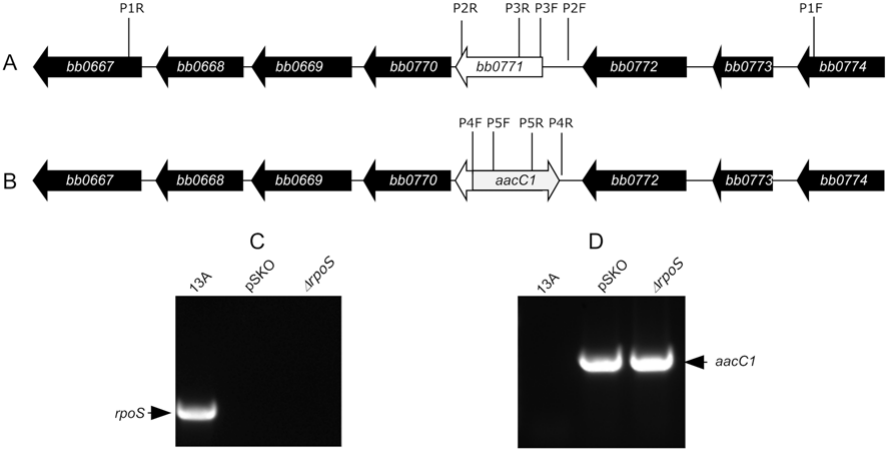
Generation of *rpoS* mutant. (A) Diagram of the *rpoS* locus (*bb0771*) and adjacent ORFs. The binding sites of six primers, i.e. P1F to P3F and P1R to P3R, are also indicated. (B) Diagram of the disrupted *rpoS* locus, showing a major portion of *rpoS* gene is replaced with the *aacC1* cassette (grey arrow). The small arrow points a residual portion of *rpoS*. The binding sites of primers P4F, P5F, P5R and P4R are also indicated. (C&D) PCR analysis of *rpoS* mutant. The 13A spirochetes, the disruption plasmid pSKO, and Δ*rpoS* were used as DNA sources and subjected to PCR amplification using the primers P3F and P3R producing an amplicon of 111 bps (C), and the primers P5F and P5R generating an amplicon of 517 bps (D).

To delete the *rpoS* gene, a disruption plasmid, pSKO, was constructed. Briefly, a 6485-bp fragment with introduced *Acc*65I and *Nhe*I restriction sites at the ends, consisting of a partial sequence of the open-reading frame (ORF) *bb0767*, the entire ORFs for *bb0768*, *bb0769*, *bb0770 bb0771* (*rpoS*), *bb0772* and *bb0773*, and a part of the ORF *bb0774*, was PCR amplified using the primers P1F and P1R (Fig. 1A; Table 1). The resulting amplicon was digested with the restriction enzymes *Acc*65I and *Nhe*I, purified using the QIAquick PCR purification kit (QIAGEN Inc., Valencia, CA), and cloned into the vector pNCO1T, as described previously [19], creating an intermediate construct, pNCO1TS. It was then used as a template to generate to a fragment with *bb0771* deleted by using the primers P2F and P2R. A gentamicin cassette (*aacC1*) was amplified from the vector pBSV2G (a gift from P. Rosa and P. Stewart) with use of the primers P4F and P4R [23]. The two amplicons were pooled, purified, digested with *Bam*HI and *Nco*I, and ligated to complete construction of pSKO.

**Table 1:** Primers used in the study a.

Primer	Sequence (5′ to 3′)^a^
P1F	ATGGTACCAGGAAGTGAAGGCAT
P1R	ACGCTAGCAAGCTGGATGCAATA
P2F	AACCATGGCAAGGCCAAAGCTTTGAG
P2R	ACGGATCCAACAGAACTTGGAATATCATC
P3F	CAGTAAGAGAACACAAGCTAATTACT
P3R	GTCGCAAGTTTGCATTTATCATCT
P4F	AACAGGATCCTAGGTAATACCCGAGCTTC
P4R	AACCATGGTCTGACGCTCAGTGGAA
P5F	TCACGGTGTTATGGAAATAG
P5R	GACTGCGAGATCATAGATATAG
P6F	AAGGATCCTTGGAGGAAATTGATGGAA
P6R	CCTCTAGACCACTGACTTACAAGTAGAC
P7F	AAGGTACCAAGATAGAGAGAGAAAAGTG
P7R	TAGGATCCTTAAGGTTTTTTTGGACTTTCT
P8F	AAGGATCCTAAATTTAATAGAAGGAGGAA
P8R	AACTGCAGGTCTTGATTATCGGGCGAAGAG

a The underlined sequences are restriction enzyme sites: *Acc*65I sites (P1F and P7F), *Bam*HI sites (P2R, P4F, P6F, P7R and P8F), *Nco*I sites (P2F and P4R), a *Nhe*I site (P1R), a *Pst*I site (P8R), and an *Xba*I site (P6R).

### Deletion of the *rpoS* locus

The *B. burgdorferi* B31 13A spirochetes were grown to late-logarithmic (log) phase in Barbour-Stoenner-Kelly H (BSK-H) complete medium (Sigma Chemical Co., St. Louis, MO) at 33°C. The clone 13A was derived from a highly transformable clone, the *B. burgdorferi* B31 5A13, which harbors 20 plasmids but lacks lp25 [24]. Further loss of lp56 makes the clone 13A more transformable [25]. Approximately 10 µg of pSKO DNA was electroporated into the 13A spirochetes harvested from a 40-ml culture; resulting gentamicin-resistant clones were screened as described previously [19]. The plasmid content of resulting mutants was analyzed as previously described [26]. The replacement of *rpoS* with the *aacC1* cassette was confirmed by PCR using the primers P3F and P3R unique for *rpoS* (Fig. 1A; Table 1), and P5F and P5R specific for the *aacC1* cassette (Fig. 1B).

### *trans*-Complementation of *rpoS* mutant and modification of the mutant to simultaneously express OspC, DbpA and DbpB

**Figure 2:**
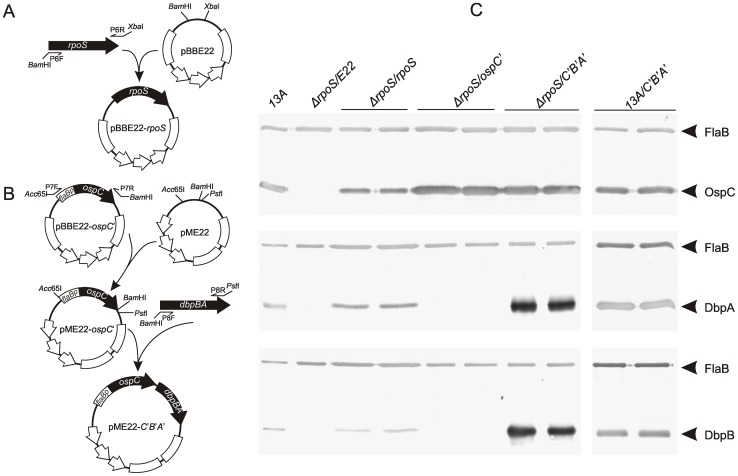
*trans-*Complementation of the *rpoS* mutant and modification of the mutant to constitutively express OspC and to simultaneously express OspC, DbpA and DbpB. (A&B) Construction of pBBE22-*rpoS* and pME22-*C*′*B*′*A*′. All restriction enzyme sites and primer binding sites used for plasmid construction are labeled. (C) Confirmation of OspC, DbpA and DbpB production by immunoblotting. The parental clone 13A and transformants Δ*rpoS/E22*, Δ*rpoS/rpoS*, Δ*rpoS/ospC′*, Δ*rpoS/C′B′A′* and 13A*/C′B′A′* were grown to late-log phase in BSK-H complete medium, harvested by centrifugation and subjected to immunoblot analyses probed with a mixture of FlaB mAb and OspC mAb (top), or mouse anti-DbpA (middle) or -DbpB sera (bottom).

Two constructs were generated as illustrated in Fig. 2. Briefly, a 1329-bp sequence, containing the entire *rpoS* gene including the upstream, coding and downstream regions, was amplified with use of the primers P6F and P6R (Table 1), digested with *Bam*HI and *Xba*I, purified, and cloned into the shuttle vector pBBE22 [27], after the vector was digested with the same enzymes, to complete construction of pBBE22-*rpoS* (Fig. 2A).

To construct pME22-*C*′*B*′*A*′, the fragment *flaBp*-*ospC* was amplified from pBBE22-*ospC′* with use of the primers P7F and P7R, digested with *Acc*65I and *Bam*HI, purified, and cloned into the shuttle vector pME22, to create pME22-*ospC*′ (Fig. 2B). pBBE22-*ospC′* and pME22 were generated in our earlier studies [19], [28]. A 1418-bp promoterless *dbpBA* operon, including the entire *dbpBA* coding region, the space between the two gene, 25 bps of upstream sequence (from the *dbpB* start codon) and 218 bps of downstream sequence (from the *dbpA* stop codon), was amplified from borrelial DNA with use of the primer P8F and P8R, digested with *Bam*HI and *Pst*I, purified, and cloned into pME22-*ospC*′, to complete construction of pME22-*C*′*B*′*A*′ (Fig. 2B). All inserts were sequenced to ensure that the inserts and their flanking sequences were as designed.

Four constructs, pBBE22, pBBE22-*rpoS*, pBBE22-*ospC′* and pME22-*C*′*B*′*A*′, were electroporated into the *rpoS* mutant; resulting transformants were identified as previously described [25]. pME22-*C*′*B*′*A*′ was also electroporated into the parental clone 13A as a control. Plasmid analyses were performed as described in our earlier study [26]. Restoration of OspC, DbpA and DbpB expression due to introduction of pBBE22-*rpoS*, overexpression of OspC due to introduction of pBBE22-*ospC′*, and simultaneous overexpression of OspC, DbpA and DbpB due to introduction of pME22-*C*′*B*′*A*′ were confirmed by immunoblot analyses, performed as described in our earlier studies [19], [25].

### Growth rate estimation

*B. burgdorferi* was grown at 33°C to late log phase (approximately 10^8^ cells/ml) in BSK-H medium, and then diluted to 10^5^ cells/ml with the medium. Cell numbers were determined once a day for 10 days under dark-field microscopy.

### Adaptation of *B. burgdorferi* in host-adapted mammalian environment

Host-adapted spirochetes were prepared in a dialysis membrane chamber (DMC) as described by Akins *et al* [29]. The Spectra/Por® 6 Standard Grade Regenerated Cellulose dialysis membrane with molecular weight cut-off of 8 kDa (Spectrum Laboratories Inc., Rancho Dominguez, CA) was treated with 5 mM EDTA and then autoclaved. Using the standard aseptic surgical procedure, a sterilized DMC was filled with 5 ml of 10^3^ spirochetes per ml suspended in complete BSK-H medium, and implanted into the peritonea of a Sprague-Dawley rat (6–8 weeks old; Division of Laboratory Animal Medicine at Louisiana State University, Baton rouge, LA). The DMC was harvested at different time points ranging from 2 to 10 weeks. All animal procedures described here and below were approved by the Institutional Animal Care and Use Committee at Louisiana State University.

### Infection study

Severe combined immunodeficient (SCID) mice on a BALB/c background (age, 4 to 6 weeks; provided by the Division of Laboratory Animal Medicine at Louisiana State University, Baton Rouge, LA) each received one single intradermal/subcutaneous injection of 10^5^ spirochetes. Inoculated animals were euthanized 1 month post-inoculation; heart, tibiotarsal joint and skin specimens were aseptically collected for spirochete culture as described previously [26].

### Estimation of tissue spirochetal load

Heart, joint, and skin specimens were harvested from infected mice and extracted for DNA. DNA was quantified for the copy numbers of *flaB* and murine actin genes by quantitative PCR (qPCR) as described previously [26]. The tissue spirochete burden was expressed as *flaB* DNA copies per 10^6^ host cells (2×10^6^ actin DNA copies).

### Quick clearance study

BALB/c SCID mice were given two intradermal/subcutaneous injections of 10^5^ spirochetes. The two inoculation sites were at least 2 cm apart. Animals were sacrificed 24 and 48 hours later; inoculation site skin tissues were harvested for spirochete isolation as described previously [26].

## Results and Discussion

### Generation of *rpoS* mutant

Thirteen gentamicin-resistant clones were obtained from electroporation of pSKO into the 13A spirochetes. Plasmid content analyses led to selection of one clone, namely, Δ*rpoS*, which had lost cp9, lp5 and lp21, in addition to lp25 and lp56. The replacement of *rpoS* with the *aacC1* cassette was confirmed by PCR using the primers unique for *rpoS* (Figure 1C), and P5F and P5R specific for the *aacC1* cassette (Figure 1D).

### *trans*-Complementation of the *rpoS* mutant and modification of the mutant to simultaneously produce OspC, DbpA and DbpB

Because the parental clone 13A and Δ*rpoS* lost lp25, the plasmid that carries the gene *bbe22* coding for a nicotinamidase essential for survival of *B. burgdorferi* in the mammalian environment, the recombinant plasmids pBBE22 and pME22, which harbor a copy of *bbe22*, were used as shuttle vectors [19], [27]. pME22 was modified from pBBE22 by replacing the restriction enzyme site *Xba*I with two sites, *Acc65*I and *Pst*I. This modification made it easy to insert a large insert into the vector. The features of the constructs are summarized in Table 2. pBBE22-*rpoS* and pME22-*C*′*B*′*A*′ were generated as illustrated in Figure 2. pBBE22-*rpoS* was constructed by cloning the full-length *rpoS* gene into pBBE22. pME22-*C*′*B*′*A*′ was created by first cloning a promoterless *ospC* gene fused with the *flaB* promoter into pME22 from pBBE22-*ospC′*, followed by inserting a promoterless *dbpBA* gene. This construct was designed in this way in order to use a single *flaB* promoter to drive constitutive OspC, DbpA and DbpB expression. pBBE22-*ospC′* were constructed in an earlier study [27], [28].

**Table 2:** Constructs and clones used in the study.

Construct or clone	Description	Source
pBBE22	pBSV2 carrying a *bbe22* copy	Reference [27]
pME22^a^	pBSV2 carrying a *bbe22* copy	Reference [19]
pBBE22-*ospC′*	pBBE22 carrying promoterless *ospC* fused with *flaB* promoter	Reference [28]
pME22-*C′B′A′*	pME22 carrying promoterless *ospC*, *dbpB* and *dbpA* fused with *flaB* promoter	This study
13A	Cloned from the *B. burgdorferi* B31 5A13	Reference [25]
Δ*rpoS*	*rpoS* mutant	This study
Δ*rpoS/rpoS*	Δ*rpoS* receiving pBBE22 carrying a wild-type *rpoS* copy	This study
Δ*rpoS/E22*	Δ*rpoS* receiving pBBE22	This study
Δ*rpoS/ospC′*	Δ*rpoS* expressing *ospC* driven by *flaB* promoter	This study
Δ*rpoS/C′B′A′*	Δ*rpoS* expressing *ospC*, *dbpB* and *dbpA* driven by *flaB* promoter	This study

a pME22 was modified from pBBE22 by removing the *bbe22* copy from the *Acc*65I site to the *Aat*II in order to make the *Acc*65I site available for cloning [19].

The four constructs were electroporated into Δ*rpoS*. pME22-*C*′*B*′*A*′ was also introduced into the parental clone 13A. Between 13 and 23 transformants were obtained from transformation with each construct. Plasmid analyses led to identification of one or two clones receiving each construct. These nine clones had the same plasmid content as Δ*rpoS*, which lost cp9, lp5, lp21, lp25 and lp56. Restoration of OspC, DbpA and DbpB expression resulting from transformation was confirmed by immunoblotting. As shown in Figure 2C, the clone Δ*rpoS/E22* didn't express any of the three surface lipoproteins as it received the empty plasmid pBBE22. As expected, transformation with the construct pBBE22-*rpoS* restored the Δ*rpoS* mutant with production of all the three lipoproteins. As the function of pBBE22-*ospC′* to drive OspC expression was already confirmed in our previous study [28], introduction of this construct, also as expected, resulted in abundant OspC production. This was the first time a single promoter was used to drive expression of three fused genes in *B. burgdorferi*. As designed, pME22-*C*′*B*′*A*′ did successfully confer the Δ*rpoS* mutant with constitutive production of OspC, DbpA and DbpB (Figure 2C). As a control, pME22-*C*′*B*′*A*′ was introduced into 13A. Although the parental strain contained the normal *rpoS* gene, whose product was expected to drive active production of the three as well as other RpoS-dependent lipoproteins, introduction of the construct did result in high levels of OspC, DbpA and DbpB production as it did in the *rpoS* mutant, probably because of the space limitation on the spirochetal surface where the three antigens have to share with other lipoproteins.

### Simultaneous expression of OspC, DbpA and DbpB doesn't alter *in vitro* growth

**Figure 3:**
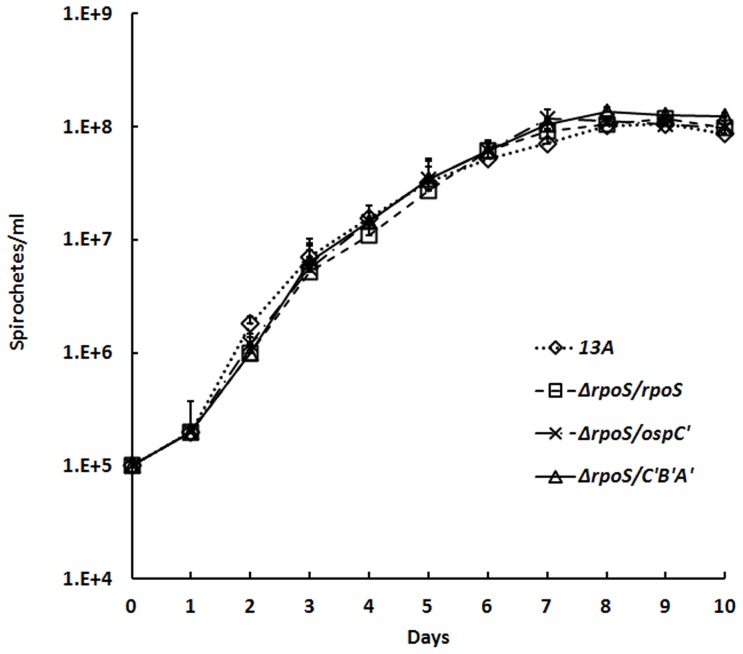
Modification of the *rpoS* mutant to constitutively produce surface lipoproteins doesn't alter its growth rate. The parental clone 13A and transformants Δ*rpoS/rpoS*, Δ*rpoS/ospC′* and Δ*rpoS/C′B′A′* were cultured in BSK-H at 33°C. Cell numbers were determined once a day for 10 days under dark-field microscopy.

We didn't notice any growth defects of the transformants during selection processes. Nevertheless, to rule out any possibility of growth defects resulting from modification of simultaneously constitutive expression of the lipoproteins, we carefully examined *in vitro* growth rates. As shown in Figure 3, all the three examined transformants, Δ*rpoS/rpoS*, Δ*rpoS/ospC′* and Δ*rpoS/C′B′A′*, produced similar growth curves as the parental clone 13A.

### Simultaneous expression of OspC, DbpA and DbpB fails to restore the *rpoS* mutant with infectivity

To examine if abundant production of the three critical virulence factors is able to overcome the absence of RpoS, groups of six SCID mice were inoculated with a single intradermal/subcutaneous dose of 10^5^ organisms of the clone Δ*rpoS/E22*, Δ*rpoS/rpoS*, Δ*rpoS/ospC′*, Δ*rpoS/C′B′A′* and 13A*/C′B′A′*. Immunodeficient mice were used because constitutive expression of the three surface lipoproteins or even OspC alone may lead to clearance by specific antibodies induced during infection of immunocompetent mice [28]. Mice were sacrificed 1 month post-inoculation; heart, tibiotarsal joint and skin specimens were harvested for spirochete culture. All of the six mice that received Δ*rpoS/rpoS* were infected, demonstrating that the Δ*rpoS* was fully competent via complementation with a wild-type *rpoS* gene (Table 3). In contrast, none of the six mice that were inoculated with the clone Δ*rpoS/E22* produced a positive tissue, a result that is consistent with previous studies showing that RpoS is essential for mammalian infection. None of the 12 mice that were challenged with the clone Δ*rpoS/ospC′* or Δ*rpoS/C′B′A′* produced a positive culture, indicating that either constitutive production of OspC or simultaneously abundant expression of OspC, DbpA and DbpB is unable to override the requirement for RpoS in murine infection.

**Table 3:** Simultaneous expression of OspC, DbpA and DbpB was unable to restore the *rpoS* mutant with infectivity a.

	No. of cultures positive/total specimens examined				No. of mice infected/total mice inoculated
Clone	Heart	Joint	Skin	All sites	
Δ*rpoS/rpoS*	6/6	6/6	6/6	18/18	6/6
Δ*rpoS/E22*	0/6	0/6	0/6	0/18	0/6
Δ*rpoS/ospC′*	0/6	0/6	0/6	0/18	0/6
Δ*rpoS/C′B′A′*	0/6	0/6	0/6	0/18	0/6
13A*/C′B′A′*	6/6	6/6	6/6	18/18	6/6

a Groups of six SCID mice were inoculated with 10^5^ organisms of the transformant Δ*rpoS/rpoS*, Δ*rpoS/E22*, Δ*rpoS/ospC′*, Δ*rpoS/C′B′A′* or 13A*/C′B′A′* and sacrificed 1 month later. Heart, tibiotarsal joint and skin specimens were harvested for spirochete culture.

**Figure 4:**
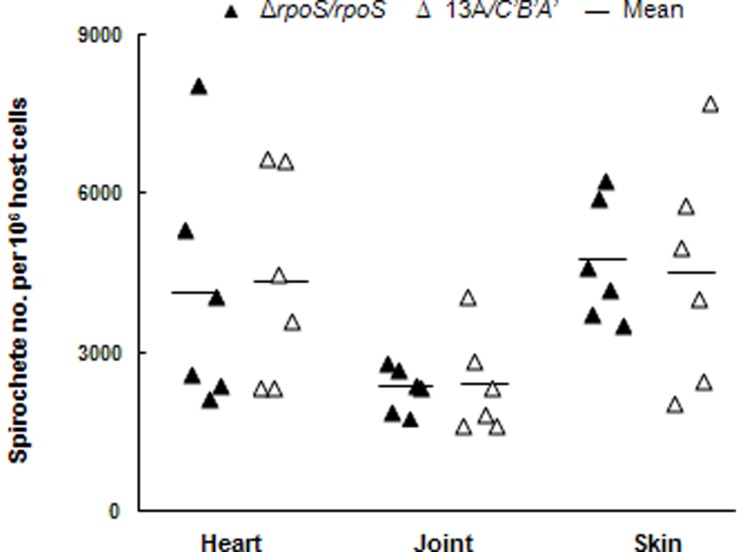
Simultaneously constitutive expression of OspC, DbpA and DbpB doesn't significantly alter tissue colonization in SCID mice. Groups of six BALB/c SCID mice were infected either with transformant Δ*rpoS/rpoS* or 13A*/C′B′A′* for 1 month. DNA samples were prepared from the heart, joint and skin specimens and analyzed for spirochete *flaB* and murine actin DNA copies by qPCR. The data are expressed as spirochete numbers per 10^6^ host cells.

All the mice that received 13A*/C′B′A′* produced culture-positive tissues. DNA samples were prepared from the heart, joint and skin specimens of infected mice and analyzed for tissue bacterial loads. As shown in Figure 4, simultaneously constitutive expression of the three lipoproteins didn't significantly alter tissue colonization of the parental spirochetes. To rule out the possibility that mutants might have been selected, spirochetes were isolated from mice that were inoculated with the transformant 13A*/C′B′A′* and further characterized. PCR amplification confirmed the presence of the introduced construct pME22-*C*′*B*′*A*′ and immunoblotting showed high levels of OspC, DbpA and DbpB expression (data not shown), demonstrating that pME22-*C*′*B*′*A*′ was well maintained during murine infection.

### Simultaneous expression of OspC, DbpA and DbpB is unable to protect the *rpoS* mutant from quick clearance

A previous study showed increasing production of DbpA driven by the *flaB* promoter reduces the ID_50_ but severely impairs dissemination, probably because the modification enhances the interaction of the pathogen with host decorin [30]. This led us to speculate that increased production of the lipoproteins, especially DbpA and DbpB, may impair dissemination and restrict spirochetes at the inoculation site. To rule out this possibility, groups of six SCID mice were given two intradermal/subcutaneous injections of 10^5^ spirochetes of the clone Δ*rpoS/rpoS*, Δ*rpoS/ospC′* and Δ*rpoS/C′B′A′*. Animals were euthanized at 24 or 48 hours later; inoculation site skin specimens were harvested for spirochete culture. As a positive control, the Δ*rpoS/rpoS* bacteria were consistently grown from all of the 12 inoculation sites harvested at 24 and 48 hours post-inoculation (Table 4). In contrast, none of the 24 sites from the 12 remaining mice produced a positive culture, indicating quick clearance of bacteria from the inoculation site.

**Table 4:** Simultaneous expression of OspC, DbpA and DbpB was unable to protect the *rpoS* mutant from quick clearance a.

Clone	No. of sites positive/Total no. of sites examined at post-inoculation hours	
	24 hours	48 hours
Δ*rpoS/rpoS*	6/6	6/6
Δ*rpoS/E22*	0/6	0/6
Δ*rpoS/ospC′*	0/6	0/6
Δ*rpoS/C′B′A′*	0/6	0/6

a Groups of six SCID mice each received two intradermal/subcutaneous injections of the transformant Δ*rpoS/rpoS*, Δ*rpoS/*E22, ΔrpoS*/ospC′* or Δ*rpoS/C′B′A′*. Approximately 10^5^ organisms were administered in each inoculation; two inoculation sites were at least 2 cm apart. Three animals from each group were euthanized at 24 and 48 hour post-inoculation; skin specimens were harvested from inoculation sites and cultured for spirochetes in BSK-H complete medium.

The remaining concern, albeit minor, was whether quick clearance of bacteria resulted from longtime, intensive genetic manipulation of *B. burgdorferi*, a process that potentially attenuates the pathogen. To rule out this possibility, the transformants Δ*rpoS/rpoS*, Δ*rpoS/ospC′* and Δ*rpoS/C′B′A′* were first grown in DMC for 2, 4 and 10 weeks. The purpose to add this treatment was not to alter OspC, DbpA and DbpB expression levels, but to give spirochetes time to adapt to the mammalian environment. Resulting host-adapted *B. burgdorferi* was inoculated into the skin of SCID mice. Animals were euthanized at 24 or 48 hours later; inoculation site skin specimens were harvested for spirochete culture. As a positive control, the Δ*rpoS/rpoS* bacteria were consistently grown from all of the 12 inoculation sites harvested at 24 and 48 hours post-inoculation (data not shown). In contrast, none of the 24 sites from the remaining 12 mice produced a positive culture. The study allowed us to conclude that simultaneous expression of OspC, DbpA and DbpB is unable to protect the *rpoS* mutant from quick clearance in murine skin.

Ten plus years of intensive investigation resulted in no additional critical RpoS-dependent virulence genes to be found, leading to a speculation whether the regulatory network regulates unknown essential virulence factors. Simultaneous expression of OspC, DbpA and DbpB is unable to restore the *rpoS* mutant with infectivity or to protect from quick clearance. Our study clearly demonstrates that RpoS controls essential virulence factors that remain to be identified.
